# Efficacy of water pressure method for colorectal endoscopic submucosal dissection: Propensity-score matching analysis

**DOI:** 10.1055/a-2544-2654

**Published:** 2025-04-04

**Authors:** Kumiko Kirita, Teppei Akimoto, Masahiro Niikawa, Keiichiro Yoshikata, Shun Nakagome, Tsugumi Habu, Yumiko Ishikawa, Eriko Koizumi, Kazutoshi Higuchi, Hiroto Noda, Takeshi Onda, Jun Omori, Naohiko Akimoto, Osamu Goto, Shunji Fujimori, Katsuhiko Iwakiri

**Affiliations:** 1 38626 Department of Gastroenterology, Nippon Medical School Chiba Hokusoh Hospital, Inzai-city, Japan; 2 26367 Department of Gastroenterology, Nippon Medical School, Bunkyo-ku, Japan

**Keywords:** Endoscopy Lower GI Tract, Colorectal cancer, Polyps / adenomas / ..., Endoscopic resection (polypectomy, ESD, EMRc, ...)

## Abstract

**Background and study aims:**

Technical difficulty of endoscopic submucosal dissection (ESD) for colorectal neoplasms has not been resolved. The water pressure method (WPM) is a helpful technique for overcome difficulties with colorectal ESD. We evaluated the efficacy and safety of ESD with WPM (WPM-ESD) for colorectal neoplasms compared with conventional ESD (C-ESD).

**Patients and methods:**

This was a single-center, retrospective, observational study. Three hundred and eleven colorectal lesions were allocated into the WPM-ESD and the C-ESD groups, which were compared before and after propensity score matching. The main outcomes were procedure time, proportion of R0 resection, and incidence of adverse events (AEs) in the two groups.

**Results:**

The WPM-ESD and C-ESD groups were allocated 134 and 177 lesions, respectively, and propensity score matching analysis created 92 matched pairs. Mean procedure time was significantly shorter in the WPM-ESD group (49 ± 26 vs. 58 ± 42 min, *P* = 0.032). All lesions were resected in one piece and there was no difference in the proportion of en bloc resection (100% vs. 100%) and R0 resection (92% vs. 96%, *P* = 0.536) or in incidence of intraoperative perforation (2.2% vs. 2.2%) in the two groups.

**Conclusions:**

WPM for colorectal ESD may shorten procedure time compared with C-ESD without increasing AEs.

## Introduction

Endoscopic submucosal dissection (ESD) is one of the minimally invasive treatments for superficial colorectal neoplasms because ESD has a high probability of complete resection en bloc, even for large neoplasms that are difficult to treat using endoscopic mucosal resection (EMR) 1 2 . However, issues with technical difficulty of ESD and risk of adverse events (AEs) associated with it have not been resolved.

Previous reports have indicated the efficacy of underwater or under-saline solution therapeutic endoscopy, such as underwater EMR 3 4 5 6 7 8 9 or underwater ESD 10 11 12 13 14 15 16 17 18 19 20 21 . Compared with carbon dioxide (CO _2_ ) insufflation, underwater conditions provide an endoscopic field of view at a lower intraluminal pressure and with a buoyancy effect, which facilitates colorectal ESD. A water pressure method (WPM), in which water pressure created by the waterjet function of an endoscope and under-saline condition facilitates insertion of the endoscope tip under the mucosal flap, has been reported to contribute to favorable results in duodenal ESD, which is a difficult and challenging procedure 13 17 . Reports exist of randomized controlled studies and meta-analyses of underwater EMR and conventional EMR for colorectal lesions 5 6 7 8 9 . A few studies have investigated the usefulness of underwater ESD or WPM for colorectal lesions compared with conventional ESD (C-ESD) 18 20 21 . The present study aimed to investigate efficacy and safety of ESD with WPM (WPM-ESD) for colorectal neoplasms compared with conventional ESD.

## Patients and Methods

### Study design and patients

In all, 291 patients with 311 colorectal lesions underwent ESD at Nippon medical school Chiba Hokusoh Hospital from August 2016 to December 2022. All ESD procedures were performed by seven endoscopists in this unit. WPM-ESD was introduced in April 2020. C-ESD was also performed after introduction of WPM-ESD. The decision to use WPM was made by the operators for each case. Traction devices were not used during the study period because they were not available in the hospital.

**Fig. 1:**
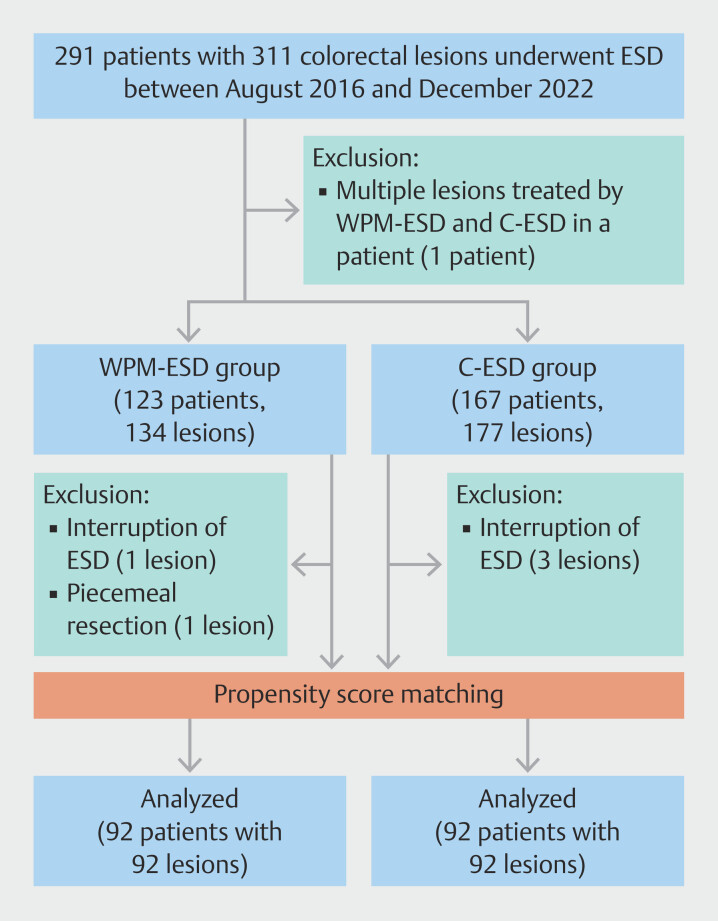
Flowchart of patients and lesions enrollment for a per-patient and a per-lesion analysis and collected lesions after adjustment of these characteristics using propensity score matching. ESD, endoscopic submucosal dissection; WPM-ESD, water pressure method ESD; C-ESD, conventional ESD.

In this study, two populations were established for per-patient and per-lesion analyses. The per-patient analysis included all but one patient, who had two lesions resected by WPM-ESD and C-ESD for each lesion. In total, 290 patients with 309 lesions were enrolled. For per-lesion analysis, five patients with five lesions in which ESD was interrupted or converted to piecemeal mucosal resection were excluded. In total, 306 lesions in 286 patients were enrolled. In addition, propensity score matching analysis was performed to adjust for lesion characteristics between the two groups ( Fig. 1 ).

All lesions were diagnosed endoscopically as being larger than 20 mm in diameter node-negative intramucosal carcinomas or as having fibrosis and were classified as appropriate lesions for treatment with ESD based on Japan Gastroenterological Endoscopy Society guidelines for colorectal ESD 22 . All patients were informed and agreed to the need for treatment and its risks and benefits before the procedures were performed and provided written informed consent to undergo ESD. This study was approved by the medical ethical committees of Nippon Medical School Chiba Hokusoh Hospital (Approval No. 952–2).

### Definition

Degree of submucosal fibrosis was classified as follows: F0, no fibrosis, which manifested as a blue transparent layer; F1, mild fibrosis, which appears as a white web-like structure in the blue submucosal layers; and F2, severe fibrosis, which appears as a white muscular structure without a blue transparent layer in the submucosal layers 23 .

Operators consisted of three experts and four non-experts. Endoscopists with experience of 50 or more colorectal ESD cases were defined as experts and endoscopists with experience of 49 or less colorectal ESD cases were defined as non-experts regardless of their experience with ESD cases on other organs. En bloc resection was defined as resection of a whole tumor in a single piece and R0 resection was defined as en bloc resection with lateral and vertical margins of the specimens histologically free of tumor cells. Procedure time was defined as time from the start of submucosal injection to removal after dissection. Perforation during ESD was defined as obvious transmural perforation such as a finding of extraluminal contents outside the intestinal tract through a hole during the procedure. Delayed perforation was defined as presence of free air on abdominal radiographs at least 12 hours after the procedure. Delayed bleeding was defined as having bloody stools after the procedure and need for endoscopic hemostasis regardless of its outcome. Postoperative fever was defined as ≥ 38°C at least 12 hours after the procedure.

### Endoscopic submucosal dissection

Patients were hospitalized the day before ESD and underwent standard bowel preparation.

All procedures were performed by seven endoscopists in this unit using a waterjet system-equipped colonoscope (PCF-Q260AI, PCF-H290I or PCF-H290TI; Olympus Co. Ltd, Tokyo, Japan) or an upper gastrointestinal endoscope (GIF-Q260J or GIF-H290T; Olympus Co. Ltd, Tokyo, Japan) for rectal lesions under insufflation with CO _2_ or under normal saline conditions. A disposable, tapered, small-caliber tip transparent hood (DH-29CR or DH-30CR; Fujifilm, Tokyo, Japan) was mounted on the tip of the endoscope.

A 1.5-mm DualKnife J (KD-655Q; Olympus Medical System, Tokyo, Japan) was mainly used for the surgical procedure. Minor bleeding during ESD was controlled by placing the closed tip of the device into the bleeding point; however, when we encountered spurting bleeding or thick arteries that could cause massive bleeding, we used hemostatic forceps (Coagrasper (FD-411QR); Olympus Medical System, Tokyo, Japan). A high-frequency electrosurgical generator (VIO3; ERBE Elektromedizine, Tubingen, Germany) was mainly used in three modes: dry cut mode (effect 2.3) for mucosal incision, swift coagulation mode (effect 3.5) for submucosal dissection, and spray coagulation mode (effect 1.2) for hemostasis using the DualKnife J. For massive bleeding, the Coagrasper was used with soft coagulation mode (effect 6.0).

We performed ESD under conscious sedation and using CO _2_ insufflation. Generally, 10% glycerin solution (Glyceol; Chugai Pharmaceutical Co. Ltd, Tokyo, Japan) containing epinephrine was used for submucosal injection. For difficult cases, such as poor submucosal layer lifting because of fibrosis, 0.4% sodium hyaluronate (Mucoup; Seikagaku Industry, Tokyo, Japan) was used as needed.

Patients with favorable physical examination, blood test, and x-ray results could drink water on postoperative day (POD) 1 and could have soft food beginning on POD 2. Patients with noticeably bloody stools requiring endoscopic hemostasis were defined as having delayed bleeding. Delayed perforation was defined as emergence of abdominal pain and free air in the abdominal space absent intraoperative perforation on imaging.

### Water pressure method

Procedure of endoscopic submucosal dissection with water pressure method. An endoscope is mainly used in the forward view. Submucosal injection is performed on the oral side of the lesion and a half circumferential mucosal incision is made. The lumen is filled with saline solution and submucosal injection is performed on the anal side. The transparent hood is gently placed on the mucosa of the anal side and mucosal incision is made. Buoyancy floats the edge of incised mucosa and the submucosa is dissected two to three times, tracing below the mucosal edge. The transparent hood is inserted under the mucosal flap by opening the submucosal space using waterjet function of the endoscope. Submucosal dissection is continued in underwater conditions and the lesion is removed.Video 1

**Fig. 2:**
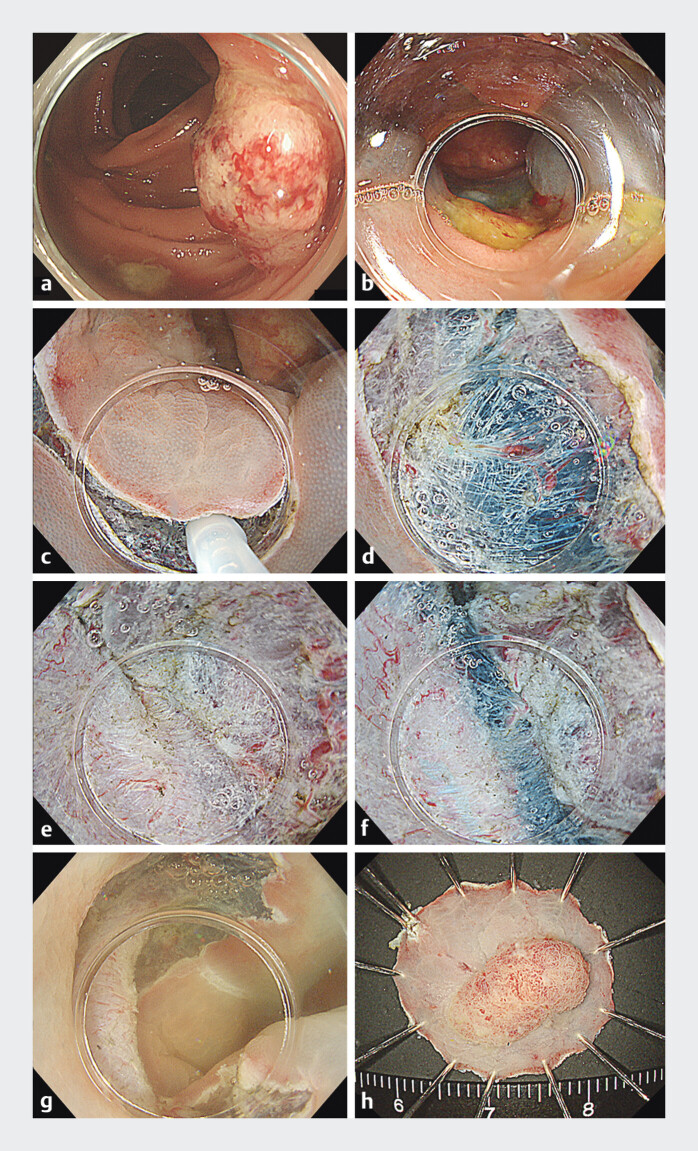
ESD with water pressure method for colorectal neoplasm. **a** Type 0-Is lesion 15 mm in diameter in the ascending colon. **b** The lesion shows non-lifting sign after submucosal injection. **c** In underwater conditions, the mucosal edge floats and the submucosa is observed clearly after mucosal incision. **d** Insertion of the transparent hood under the mucosal flap. **e** Severe fibrotic tissue is observed in submucosa. **f** Additional submucosal injection lifts the lesion and dissection of fibrotic tissue is completed. **g** The lesion is removed without perforation. **h** En bloc resection is achieved.

A typical case of WPM-ESD is presented in the endoscopic images and video ( Fig. 2 , Video 1 ). For this technique, we first performed submucosal injection and then made a semicircular mucosal incision on the oral side under CO _2_ insufflation or in underwater conditions. After mucosal incision of the oral side, we sucked all of the air out and filled the lumen with normal saline solution using the waterjet function of an endoscope and made a mucosal incision on the anal side. Consequentially, the submucosal space below the mucosal edge was opened by the effects of buoyancy and active water pressure by the waterjet function of the endoscope, which facilitated insertion of the transparent hood under the mucosal flap. WPM is defined as this process and submucosal dissection that was continued in underwater conditions. During submucosal dissection, buoyancy of the dissected specimen acted as countertraction and water pressure made it easier to visualize the lateral edge of the dissection layer, facilitiated efficient dissection of the submucosal layer 17 19 . Finally, after the lesion was removed, prophylactic hemostasis of the vessels that had post-ESD mucosal defects was performed.

### Outcome measures and propensity-score matching

In the present study, the primary outcome was procedure time for ESD, defined as time from submucosal injection to removal of lesions. Secondary outcomes were en bloc resection rate, incidence rate of intraoperative perforation, delayed bleeding and delayed perforation, and fever defined as > 38.0°C after ESD. In addition, the postoperative course was evaluated in terms of inflammatory markers (serum C-reactive protein [CRP] levels, white blood cell count [WBC]) on POD 1, hemoglobin levels on POD 1, and hospitalization period. We compared these outcomes between the WPM-ESD group and the C-ESD group.

To investigate efficacy of WPM-ESD, the two groups were compared after propensity score matching to control for factors that might influence ESD treatment outcomes and AEs. In a per-lesion analysis of the population ( Fig. 1 ), potential factors related to lesion characteristics were assessed and univariate analysis was performed with the explanatory variables of location, morphology, tumor size, muscle layer retraction, and pathology. To minimize inherent bias, the two groups were matched in a 1:1 ratio with 92 patients in each group and propensity score matching was used to adjust for the two covariates determined by the univariate analysis, tumor location and tumor size.

### Statistical analyses

Statistical analyses were performed using the SPSS statistical software package version 25 (IBM, New York, United States). Pearson’s chi-square test or Fisher exact test was used to analyze categorical variables. Mann-Whitney U test was used for unpaired data to determine differences in means between two groups. *P* < 0.05 was considered statistically significant.

## Results

### Analysis before propensity score matching

In per-patient analysis, 123 patients and 167 patients were allocated to the WPM-ESD and C-ESD groups, respectively. In per-lesion analysis, 134 lesions and 177 lesions were allocated to the WPM-ESD and C-ESD groups, respectively ( Fig. 1 ). Table 1 shows characteristics of the patients and lesions and the experience of operators. The proportion of rectal lesions to colonic lesions in the WPM-ESD group was lower than in the C-ESD group (10% vs. 36%, *P* < 0.001) and mean lesion size in the WPM-ESD group was smaller than that in the C-ESD group (23.3 ± 9.3 mm vs. 26.8 ± 12.2 mm, *P* = 0.005). No significant differences were found in proportion of en bloc resection, R0 resection, or incidence of AEs between the two groups. However, mean procedure time was significantly shorter in the WPM-ESD group than in the C-ESD group (49.6 ± 27.9 min vs. 64.6 ± 51.6 min, *P* = 0.001).

**Table:** **Table 1** Characteristics of patients and lesions between WPM-ESD and C-ESD before and after propensity score matching.

	All patients			Propensity score-matched patients		
	WPM-ESD (123 patients 134 lesions)	C-ESD (167 patients 177 lesions)	*P* value	WPM-ESD (92 patients 92 lesions)	C-ESD (92 patients 92 lesions)	*P* value
Patients	N = 123	N = 167		N = 92	N = 92	
Age, mean ± SD, years	71 ± 10	69 ± 11	0.124	70 ± 10	71 ± 10	0.447
Sex, n (%)			0.111			0.13
Male	69 (56%)	111 (66%)		51 (55%)	62 (67%)	
Female	54 (44%)	56 (34%)		41 (45%)	30 (33%)	
Antithrombotic agents, n (%)	43 (35%)	64 (38%)	0.304	35 (38%)	39 (42%)	0.652
Lesions	N = 134	N = 177		N = 92	N = 92	
Tumor size, mean ± SD, mm	23.3 ± 9.3	26.8 ± 12.2	0.005	23.1 ± 7.5	23.9 ± 8.2	0.475
Location, n (%)			< 0.001			NA
Colon	120 (90%)	113 (64%)		80 (87%)	80 (87%)	
Rectum	14 (10%)	64 (36%)		12 (13%)	12 (13%)	
Morphology, n (%)			0.56			0.412
Protruded type	28 (21%)	57 (32%)		19 (21%)	27 (29%)	
LST-G	50 (37%)	73 (41%)		33 (36%)	36 (39%)	
LST-NG	56 (42%)	47 (27%)		40 (44%)	29 (32%)	
Fibrosis, n (%)			0.349			0.161
F0	110 (82%)	137 (77%)		79 (86%)	72 (78%)	
F1	17 (13%)	30 (17%)		11 (12%)	18 (20%)	
F2	7 (5%)	10 (6%)		5 (5.4%)	2 (2%)	
R0 resection, n (%)	121 (91.6%)	164 (94.3%)	0.253	85 (92%)	88 (96%)	0.536
Pathology*, n (%)			0.084			0.213
adenoma or SSL	25 (19%)	45 (26%)		19 (21%)	31 (34%)	
Tis	88 (67%)	109 (63%)		64 (70%)	53 (58%)	
T1a	6 (4.5%)	12 (6.9%)		5 (5.4%)	6 (6.5%)	
T1b	12 (9.1%)	5 (2.9%)		4 (4.3%)	2 (2.2%)	
Others	1 (0.8%)	3 (1.7%)		0 (0%)	0 (0%)	
Procedure factor	N=134	N=177		N=92	N=92	
Operator, n (%)			0.283			0.172
Expert	82 (61%)	119 (67%)		52 (57%)	62 (67%)	
Non-expert	52 (39%)	58 (33%)		40 (43%)	30 (33%)	
ESD, endoscopic submucosal dissection; WPM, water pressure method; C-ESD, conventional ESD; SD, standard deviation; LST-G, laterally spreading tumor, granular type; LST-NG, laterally spreading tumor, nongranular type; POD, postoperative day; SSL, sessile serrated lesion. *Described only resected and evaluated lesions.						

### Analysis after propensity score matching

As Fig. 1 shows, excluding five lesions (2 lesions in the WPM-ESD group and 3 lesions in the C-ESD group) which could not be resected en bloc, 306 lesions were identified in the two groups before propensity score matching analysis. Propensity score matching controlled for differences in proportion of location of lesions and mean lesion size, which were significantly different between the two groups on univariate analysis. The caliper width of the matching was 0.0504. This model yielded a c-statistic of 0.786, indicating its ability to differentiate between the two groups. Consequently, propensity score matching created 92 matched pairs ( Fig. 1 ). Table 2 shows characteristics of the two groups after propensity score matching. After adjusting for lesion size and location, there were no difference in other characteristics. There were 80 colon lesions and 12 rectal lesions in both groups. Mean tumor size in the WPM-ESD group and in the C-ESD group were 23.1 ± 7.5 mm and 23.9 ± 8.2 mm, respectively ( *P* = 0.475). Of these, 52 lesions in the WPM-ESD group and 62 lesions in the C-ESD group were resected by experts. The others were resected by non-experts. Even after adjusting characteristics, mean procedure time in the WPM-ESD group was significantly shorter than that in the C-ESD group (48.7 ± 25.6 min vs. 58.1 ± 41.5 min, *P* = 0.032). There were no significant differences in proportion of en bloc resection (100% vs. 100%), R0 resection (92% vs. 96%, *P* = 0.536), and rates of perforation during ESD between the two groups (2% vs. 2%).

**Table:** **Table 2** ESD outcomes between WPM-ESD and C-ESD of before and after propensity score matching **.**

	All patients			Propensity score-matched patients		
	WPM-ESD (123 patients/134 lesions)	C-ESD (167 patients/177 lesions)	*P* value	WPM-ESD (92 patients/92 lesions)	C-ESD (92 patients/92 lesions)	*P* value
ESD outcomes						
En bloc resection, n (%)	132 (98.5%)	174 (98.3%)	0.63	92 (100%)	92 (100%)	NA
R0 resection, n (%)	121 (91.6%)	164 (94.3%)	0.253	85 (92%)	88 (96%)	0.536
Procedure time of ESD, mean ± SD, min	49.6 ± 27.9	64.6 ± 51.6	0.001	48.7 ± 25.6	58.1 ± 41.5	0.032
Perforation during ESD, n/N (%)	4/134 (3.0%)	5/177 (2.8%)	0.933	2/92 (2.2%)	2/92 (2.2%)	NA
Delayed bleeding, n (%)	5 (4.1%)	7 (4.2%)	0.957	5 (5%)	1 (1%)	0.211
Delayed perforation, n (%)	0 (0%)	0 (0%)	NA	0 (0%)	0 (0%)	NA
Fever after ESD, n (%)	7 (5.7%)	4 (2.4%)	0.146	5 (5%)	3 (3%)	0.36
Findings of blood examination on POD 1						
White blood cell counts, mean ± SD, /μL	8062 ± 2860	6751 ± 2613	< 0.001	8099 ± 2994	6842 ± 2859	0.005
Hemoglobin levels, mean ± SD, g/dL	13.5 ± 7.26	13.0 ± 1.71	0.434	13.7 ± 8.62	13.1 ± 1.72	0.474
C-reactive protein, mean ± SD, mg/dL	1.45 ± 1.95	0.60 ± 1.00	< 0.001	1.54 ± 2.06	0.58 ± 0.74	< 0.001
Hospitalization period, mean ± SD, days	5.4 ± 4.6	6.0 ± 2.5	0.212	5.7 ± 5.5	6.1 ± 2.9	0.256
C-ESD, conventional ESD; ESD, endoscopic submucosal dissection; POD, postoperative day; SD, standard deviation; SSL, sessile serrated lesion; WPM, water pressure method.						

Mean WBC count and CRP levels were significantly higher in the WPM-ESD group than in the C-ESD group (WBC 8099 ± 2994 vs. 6842 ± 2839, *P* < 0.001, CRP 1.54 ± 2.06 vs. 0.58 ± 0.74, *P* < 0.001).

## Discussion

In this study, safety and efficacy of WPM-ESD and C-ESD for colorectal tumors were compared retrospectively. Although this study was not randomized or controlled, it was a comparative study with a sample size sufficient to compare 92 pairs with matched characteristics of patients and lesions by propensity score matching analysis. Moreover, this study was the first to compare ESD outcomes considering different experiences of ESD of multiple operators. The strength of this study was that we demonstrated tha WPM-ESD shortened procedure time without decreasing resectability and increasing incidence of AEs after adjusting background factors. There have been a few reports presenting usefulness of WPM-ESD and C-ESD. Masunaga et al. revealed that WPM-ESD shortened procedure times for novice endoscopists in colorectal ESD of bovine rectum. Ozeki et al. reported that WPM-ESD shortened procedure time of ESD for colorectal lesions with submucosal fibrosis 18 . Koyama et al. reported that underwater ESD decreased incidence of post-ESD coagulation syndrome and shortened procedure time compared with C-ESD 21 . However, the numbers and experiences of the operators were not considered in these clinical retrospective studies. Two randomized controlled studies comparing ESD outcomes between WPM-ESD and C-ESD have been reported 24 25 . In both studies, ESD was performed at single center by one operator.

It has been reported that colorectal ESD difficulty is related to tumor size, tumor location, macroscopic type such as LST-NG, submucosal fibrosis and muscle-retracting sign, and operator experience 26 27 28 29 . These factors also contribute to procedure time. In this study, there were significant differences between the two groups in lesion location and tumor size; therefore, we homogenized them to allow us to equalize difficulty of treatment between WPM-ESD and C-ESD by propensity score matching as much as possible and to evaluate treatment outcomes. Moreover, this study considered the experience and skills of multiple operators.

Several factors contribute to this result. First, performing the procedure underwater with saline provides buoyancy and water pressure lifts the submucosal layer out of the muscular layer 3 . Buoyancy also works as countertraction and helps open the mucosal flap. Regardless of patient position, buoyancy can be obtained by filling the lumen with saline. Second, WPM-ESD also has the benefit of a magnifying effect from the optical zoom, and working underwater reduces reflection and fogging of the lens and clarifies the endoscopic field of view 3 . Third, an important factor is that water pressure associated with adding saline solution using the waterjet function of the endoscope helps open the submucosa, allowing accurate identification of dissection lines and lateral edges of incised mucosa even in a narrow space 17 19 . Consequently, these factors contribute to stabilizing the field of endoscopic view and creating mucosal flaps, which is an important step in ESD.

Buoyancy and water pressure in underwater conditions work as countertraction methods and facilitate colorectal ESD as described previously. Colorectal ESD using traction devises or traction methods with specially prepared string also has been reported to shorten procedure time 30 31 32 . However, these traction devises require dedicated equipment, an additional procedure for installation/removal, and additional cost for the devices. Traction methods that use specially prepared string also require time for preparation. Moreover, mechanical traction devices are difficult to remove once attached, whereas underwater conditions and water pressure are advantageous in that they can be quickly introduced and interrupted, depending on what is occurring during ESD.

Disadvantages of WPM-ESD are poor endoscopic visibility in the case of insufficient bowel preparation or massive bleeding requiring endoscopic hemostasis with hemostatic forceps. Although massive bleeding during colonic ESD is minor compared with that of gastric ESD, blood mixes with saline solution in underwater ESD, resulting a poor view of the operative field. In addition, prolonged bleeding results in submucosal hematomas, which prevent efficient submucosal dissection. Therefore, prophylactic hemostasis for visible vessels and rapid hemostasis for intraoperative bleeding are needed when WPM-ESD is performed. A clear endoscopic view in underwater conditions makes recognition of vessels easy and pre-coagulation of them more feasible.

Although no significant differences were observed between the two groups in this study regarding intraoperative perforation, postoperative perforation, postoperative fever, or period of hospitalization, CRP levels and WBC counts were higher in patients who underwent WPM-ESD than in those who underwent C-ESD. Generally, risk of bacteremia is low after colorectal EMR and ESD 33 . There also have been reports of bacteremia after colorectal ESD and CRP levels can be decreased by prophylactic administration of antibiotics 34 35 . These factors may suggest that the increased CRP levels and WBC counts associated with WPM-ESD were related to bacterial infection through wounds created by ESD. WPM-ESD involves filling the lumen with saline solution, which means that the wounds are continuously exposed to saline solution containing stool residue and enteric bacteria during the treatment. Therefore, CRP levels and WBC counts may have been higher in the WPM-ESD group in this study, although there was no clinical impact. Further study is needed in this regard.

Recently, CK Oh et al. reported on randomized controlled trial (RCT) of procedure time and dissection speed in 28 patients assigned to an underwater ESD group and a conventional ESD group. Both procedure time and dissection speed were faster with underwater ESD than with conventional ESD 24 . In contrast, Nagata et al. reported that underwater ESD did not facilitate dissection speed in colorectal ESD procedures in the overall patient population in another randomized controlled trial 25 . However, in these two single-center RCTs, there were some limitations in that the number of cases was small and a single expert performed ESD. Thus, a multicenter study and meta-analysis are warranted in the future.

There are several limitations to this study. First, it was performed in a single-center and retrospective. Second, selection bias was present: The choice between WPM-ESD and C-ESD was made by the endoscopist, resulting in differences in lesion characteristics (size and location). WPM was introduced midway through the study period whereas it would have been better to compare the WPM-ESD group and the C-ESD group for the complete term. That is because the difference in term may have caused a bias in learning curve that directly influenced the procedure time, which was not considered in this study. However, the number of cases was too small to allow for adjustment of lesion characteristics using propensity score matching. Therefore, endoscopist experience was investigated and there was no difference in the proportions of expert and non-experts between the WPM-ESD group and the C-ESD group.

## Conclusions

In conclusion, the findings from this retrospective study suggest that compared with C-ESD, WPM-ESD provides comparable resectability and may reduce procedure time for colorectal tumor resection without increasing AEs. The results also support the efficacy and safety of WPM-ESD. Based on the data from this study and previous ones, a RCT with a statistically calculated number of patients is required to demonstrate the clinical effectiveness of WPM-ESD.
